# A Novel Virtual Reality-Based System for Measuring Deviation Angle in Strabismus: A Prospective Study

**DOI:** 10.3390/diagnostics15182402

**Published:** 2025-09-20

**Authors:** Jhih-Yi Lu, Yin-Cheng Liu, Jui-Bang Lu, Ming-Han Tsai, Wen-Ling Liao, I-Ming Wang, Hui-Ju Lin, Yu-Te Huang

**Affiliations:** 1 Eye Center, China Medical University Hospital, Taichung 404327, Taiwan; 032752@tool.caaumed.org.tw; 2 Department of Information Engineering and Computer Science, Feng Chia University, Taichung 407102, Taiwanminghtsai@fcu.edu.tw (M.-H.T.); 3 Graduate Institute of Integrated Medicine, China Medical University, Taichung 406040, Taiwan; 4 Personal Medical Research Center, China Medical University Hospital, Taichung 404327, Taiwan; 5 Genetic Center, Department of Medical Research, China Medical University Hospital, Taichung 404327, Taiwan; 6 School of Chinese Medicine, China Medical University, Taichung 406040, Taiwan

**Keywords:** virtual reality, strabismus, camera rotate, target offset

## Abstract

**Background/Objectives**: To develop a new Virtual Reality (VR) system software for measuring ocular deviation in strabismus patients. **Methods**: This prospective study included subjects with basic-type exotropia (XT) and non-refractive accommodative esotropia (ET). Ocular deviation was measured using the alternate prism cover test (APCT) and two VR-based methods: target offset (TO) and a newly developed camera rotation (CR) method. **Results**: A total of 28 subjects were recruited (5 cases were excluded for preliminary testing and 5 for not meeting inclusion criteria). Among the 18 included patients, 10 (66.7%) had XT and 5 (33.3%) had ET. The median age was 21.5 years (IQR 17 to 25). The mean age was 22.3 years (range: 9–46), with 5 (27.8%) having manifest strabismus and 12 (61.1%) measured while wearing glasses. VR-based methods (TO and CR) showed comparable results to APCT for deviation angle measurements (*p* = 0.604). Subgroup analysis showed no significant differences in ET patients (all *p* > 0.05). In XT patients, both TO and CR underestimated deviation angles compared to APCT (*p* = 0.008 and *p* = 0.001, respectively), but no significant difference was observed between the two methods (*p* = 0.811). Linear regression showed CR had a stronger correlation with APCT than TO (R^2^ = 0.934 vs. 0.874). **Conclusions**: This newly developed VR system software, incorporating the CR method, provides a reliable approach for measuring ocular deviation. By shifting the entire visual scene rather than just the target, it lays a strong foundation for immersive diagnostic and therapeutic VR applications.

## 1. Introduction

Strabismus, a misalignment of the eyes, can be either congenital or acquired. While it is more commonly associated with children, the estimated incidence of adult strabismus is around 4% [1,2]. Despite its prevalence, the global shortage of pediatric ophthalmologists has become increasingly apparent in recent years, driven by a growing worldwide population and limited resident’s interest in this specialty [3]. This issue is further worsened by the unequal distribution of pediatric ophthalmologists, which limits access to care in regions with lower socioeconomic status [4]. These challenges may also contribute to the decline in the total number of strabismus procedures performed by ophthalmology residents during residency [5].

To address the challenges posed by the shortage and unequal distribution of pediatric ophthalmologists, as well as the limitations of traditional methods for diagnosing and measuring strabismus, several emerging technologies have been introduced [6,7,8,9]. These advancements, including both hardware and software system designs, aim to improve accessibility, efficiency, and accuracy in clinical practice. On the hardware side, innovations such as smartphone-based applications, smart glasses systems, and virtual reality (VR) platforms have been developed to simplify the diagnostic process and reduce dependence on operator expertise [10,11,12]. While these instruments differ in design, they share a common approach: using infrared light to generate reflections, which are captured by optical sensors and cameras. By calculating the angle between the corneal reflex and the pupil center, these systems accurately determine the gaze direction [6].

Among these hardware systems, VR offers distinct advantages. It provides a fully immersive experience, enabling more precise and realistic simulations of visual conditions and therapeutic interventions [13]. This capability allowed tailored visual environments designed for specific purposes, such as vision training and myopia management. This flexibility makes VR particularly valuable, as it can replace parts of the diagnostic process and broaden its use in various clinical and therapeutic applications.

Previous VR studies used target offset (TO) methods to measure ocular misalignment angle in strabismus patients, where the target was moved on the VR screen to simulate the prism cover effect [14,15]. Despite showing a good correlation with the alternative prism cover test (APCT) performed by experienced ophthalmologists, it is challenging to extend this method to 3D dimensions where strabismus training involves movement and head rotation.

Consequently, we designed another method named “camera rotation” (CR), which rotates the entire camera view to mimic a misaligned eye. Unlike the TO method, which moves only the target, the CR method shifts the entire visual scene, including both the background and the target. This comprehensive adjustment allows for the creation of immersive visual environments that more accurately replicate real-world conditions. Such a design not only improves the realism of the simulation but also provides the foundation for future applications tailored to specific therapeutic purposes, such as vision training and other personalized treatments.

In the present study, we conducted a prospective study to evaluate the use of commercial VR headsets with built-in eye tracking, utilizing the newly developed CR method. Our objective was to compare the measurement agreement of the CR method with the traditional APCT and the previously established TO method.

## 2. Materials and Methods

### 2.1. Study Design

This prospective case series was conducted at China Medical University Hospital (CMUH) between August 2022 and December 2023. Approval was obtained from the institutional review board of CMUH (IRB number: CMUH111-REC1-085, approved on 13 April 2023), and informed consent was obtained from all participants. Written informed consent was also obtained from the patients to publish this paper. The study followed the principles outlined in the Declaration of Helsinki and complied with internationally recognized standards for research conduct and reporting.

### 2.2. Study Populations

Patients with the diagnosis of strabismus were recruited from the ophthalmology clinic in China Medical University Hospital. To minimize confounding variables in strabismus group, only patients with basic-type intermittent exotropia or non-refractive accommodative esotropia were included. Patients with incomitant or vertical strabismus were excluded. Patients with poor visual acuity (VA), defined as best-corrected VA less than 20/40, poor concentration, ptosis, or psychological conditions such as attention deficit hyperactivity disorder that could affect test accuracy were also excluded. All patients underwent a full orthoptic assessment by a single experienced pediatric ophthalmologist (HJL), including APCT at a 6 m distance fixation.

### 2.3. VR Equipment

**Figure 1:**
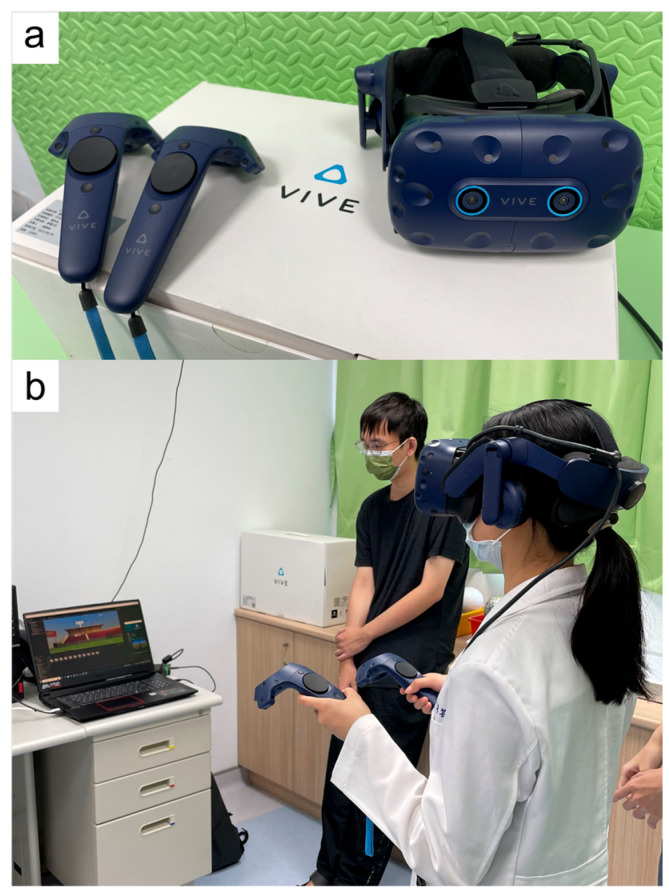
(**a**) The VR equipment setup, including the HTC VIVE Pro Eye headset and a standard controller. (**b**) The participant’s positioning during the testing session.

In this study, we used the HTC VIVE Pro Eye, a head-mounted virtual reality (VR) system developed by HTC Corporation (Taoyuan City, Taiwan). The system was released in 2019 and features dual 3.5-inch AMOLED displays with a resolution of 1440 × 1600 pixels per eye (2880 × 1600 combined), a refresh rate of 90 Hz, and a 110-degree horizontal field of view. The system supports integrated eye tracking via Tobii technology. Participants interacted using the standard VIVE controllers. The software was developed on the SteamVR platform. This device was selected for its integrated eye-tracking support, high display resolution, and compatibility with immersive software development [16]. A visual overview of the VR headset, controller, and participant testing position is shown in Figure 1.

### 2.4. Deviation Angle Measurements by VR Using Two Methods

When participants put on the VR headset, they saw a white circular ring, measuring 20 cm in diameter, positioned 6 m directly in front of them in the VR environment. Randomly, one eye would see the ring while the other eye displayed a black screen (covered). Every 3 s, we alternated covering both eyes using the VR system and observed the horizontal movement of the covered eye with an eye tracker (at the moment of switching and 0.3 s after).

**Figure 2:**
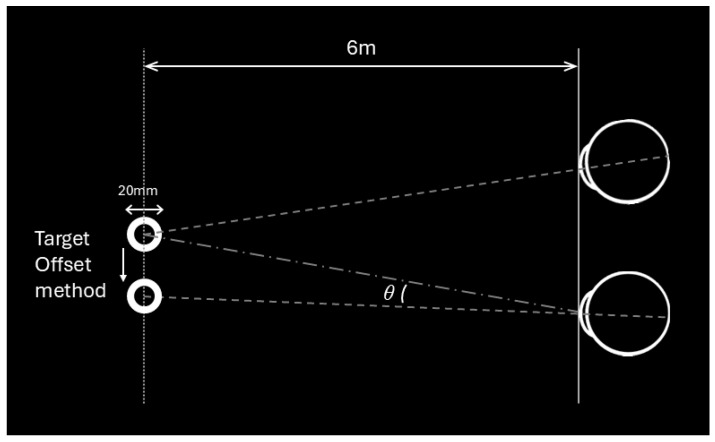
The Target Offset (TO) method involved constructing a virtual environment with two white rings, each 20 mm in diameter, placed in each eye’s view against a completely black background. These rings were positioned at a distance of 6 m from the patient. The position of the target was then adjusted using the TO method to measure the deviation angle, denoted as θ.

**Figure 3:**
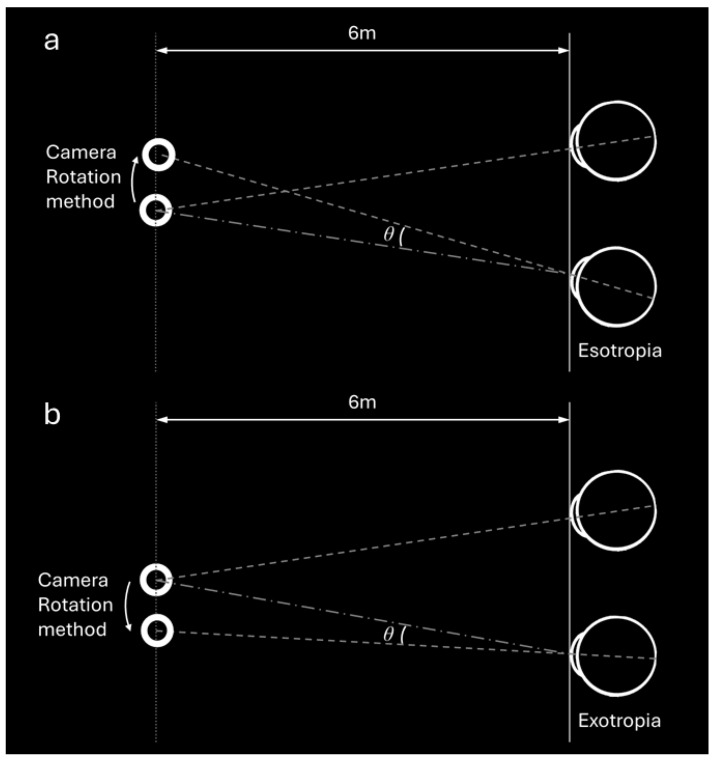
(**a**) The Camera Rotation (CR) method used the same virtual environment setup as described previously. For a patient with esotropia, the camera in front of one eye was moved temporally using the CR method. This simulated the effect of a virtual base-out prism. The rotation angle in this context was also denoted as θ. (**b**) For a patient with exotropia, the camera view of one eye was rotated outward to align with the deviated eye, simulating the effect of a virtual base-in prism. The rotation angle was similarly denoted as θ.

If there was a significant eye movement (prism error >1 prism diopter (PD)), we adjusted using two methods: First, by changing the position of the ring on the VR display (TO method) (Figure 2); second, by rotating the camera of one eye according to the eye movement direction, referred to as the camera rotation (CR) method (Figure 3a,b). In the CR method, the entire camera view, including the background and all visible elements, were rotated to align with the deviation of the eye rather than moving the target itself. For example, in an esotropic patient (Figure 3a), the camera was rotated inward to match the inward deviation of the eye, creating a corrected image that compensates for the misalignment. Conversely, for an exotropic patient (Figure 3b), the camera was rotated outward to correspond to the outward deviation of the eye. This adjustment ensured that the visual feedback provided by the VR headset reflected the patient’s eye position, allowing for precise measurement of the alignment.

### 2.5. Statistical Analysis

All statistical analyses were performed using SPSS version 22.0 (IBM Corp., Armonk, NY, USA). Continuous variables were reported as mean ± standard deviation (SD), and categorical variables as percentages. The Shapiro–Wilk test was used to assess normality of continuous variables. For group comparisons, paired *t*-tests or Wilcoxon signed-rank tests were used as appropriate. Kendall’s coefficient of concordance was used to evaluate inter-rater agreement. The coefficient of determination (R^2^) was calculated from linear regression models to assess the agreement between VR-based methods and APCT. All statistical tests were two-sided, and a *p*-value < 0.05 was considered statistically significant.

## 3. Results

### 3.1. Baseline Characteristic of the Test Subjects

Twenty-eight cases were included in the study, with 5 cases used for preliminary testing and parameter adjustments, and thus not included in the statistical analysis. Three cases were excluded due to the eye-tracking system’s headset failing to accurately capture the pupil’s position. Two cases were excluded because their deviation angle was too large (>60 PD), exceeding the VR system’s upper limit. The remaining 18 cases were included in the final analysis.

The mean age of the analyzed subjects was 22.3 years, ranging from 9 to 46 years. The median age was 21.5 years (IQR 17 to 25). Of these, 77.8% were female, and 22.2% were male. Measurements were obtained while wearing glasses in 61.1% of the cases. Among the included participants, 33.3% (*n* = 6) were diagnosed with esotropia (ET), and 66.7% (*n* = 12) had exotropia (XT). Manifest strabismus was present in 27.8% (*n* = 5) of the subjects. The baseline characteristics are shown in Table 1.

### 3.2. Deviation Angle Measurements by APCT and VR Using Two Methods

The mean deviation angle measured by APCT was 16.00 ± 32.23 PD. Using the TO method in VR, the mean deviation angle was 7.67 ± 29.54 PD, while the CR method produced a mean deviation angle of 6.22 ± 32.36 PD. No statistically significant differences were found among the three methods (*p* = 0.604).

Subgroup analysis showed that in esotropic patients (*n* = 6), the paired differences among TO, CR, and APCT measurements were comparable, with TO-CR at 5.12 ± 11.69 PD (*p* = 0.328), APCT-TO at 3.83 ± 11.44 PD (*p* = 0.499), and APCT-CR at 9.00 ± 9.38 PD (*p* = 0.066). These findings indicated no significant differences among the three methods in this group. In exotropic patients (*n* = 12), both TO and CR methods underestimated the deviation angle compared to APCT, with APCT-TO at 10.58 ± 11.23 PD (*p* = 0.008) and APCT-CR at 10.17 ± 8.19 PD (*p* = 0.001). The paired difference between TO and CR was −0.43 ± 5.88 PD, showing no significant difference between these two methods (*p* = 0.811). The data are summarized in Table 2.

**Figure 4:**
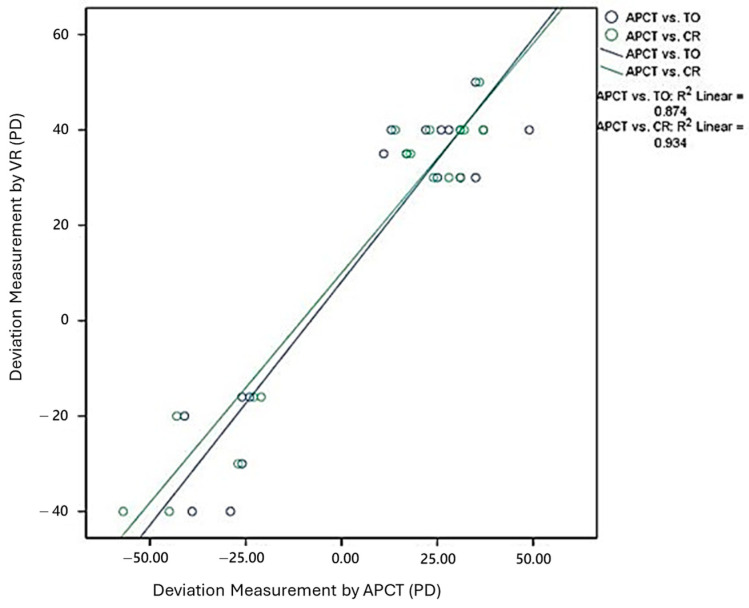
Linear regression analysis comparing deviation angles measured using (A) Camera Rotation (CR) and (B) Target Offset (TO) methods with those from the alternate prism cover test (APCT). Each point represents an individual subject. The green regression line reflects the CR method (R^2^ = 0.934), and the blue regression line reflects the TO method (R^2^ = 0.874). The closer clustering of green points along the line suggests better agreement with APCT.

In the scatter plot shown in Figure 4, the green circles represent the deviation angles measured using APCT and CR, while the blue circles represent those measured using APCT and TO. The green circles are more closely clustered along the regression line, indicating a stronger agreement between CR and APCT. In contrast, the blue circles show a wider spread, suggesting less consistent alignment between TO and APCT. Linear regression analysis showed that CR had a higher coefficient of determination (R^2^ = 0.934) compared to TO (R^2^ = 0.874), indicating better agreement between CR and APCT measurements. In addition, Pearson correlation coefficients were r = 0.966 for CR vs. APCT and r = 0.935 for TO vs. APCT, both statistically significant (*p* < 0.001). Bland–Altman analysis was performed to assess agreement between APCT and the two VR-based methods. For TO vs. APCT, the mean difference was −8.33 PD, with 95% limits of agreement from −30.75 to +14.08 PD. For CR vs. APCT, the mean difference was −9.78 PD, with 95% limits of agreement from −26.13 to +6.57 PD. Although the CR method showed slightly larger bias compared to the TO method, it had narrower 95% limits of agreement, suggesting more consistent measurements and better overall agreement with APCT.

## 4. Discussion

In this present study, we assessed the accuracy of a VR system using a commercial headset with a built-in eye-tracking function and a newly developed CR method. This VR system demonstrated the ability to provide comparable ocular misalignment measurements to the previous TO method, with a stronger correlation to APCT, which is considered the golden standard [11,12]. Our results showed no significant differences among the three methods overall, but subgroup analysis revealed distinct trends: the CR and TO methods showed comparable accuracy in esotropic patients, whereas both methods underestimated deviation angles in exotropic patients when compared to APCT.

Currently, several VR-based systems are being actively investigated, each with its strengths and limitations [12,14,15,17,18]. Nesaratnam et al. reported on 3 cases comparing the efficacy of the Oculus Rift VR headset to the traditional Lees screen test, achieving good correspondence between the two methods [17]. However, this study only provided descriptive results without further quantification. Miao et al. and Yeh et al. utilized FOVE and VIVE Pro Eye headsets, respectively, to test for ocular deviation. Both studies reported good agreement between VR-based measurements and those made by trained ophthalmologists (mean difference less than 0.7° and ICC = 0.897, respectively) [14,15]. However, their reliance on TO methods limited their applicability to dynamic 3D training programs.

Martinez Mori et al. developed a VR-simulated alternate cover test using the Olleyes VisuALL ETS system, which showed moderate correlation with the traditional APCT (r = 0.42) but greater consistency in esotropic cases (r = 0.74). However, variability in exotropic measurements and low diagnostic sensitivity (27.6%) highlighted areas for improvement [12]. Similarly, Nixon et al. introduced the STARE system for automated strabismus screening. While it demonstrated excellent specificity (100%) and an AUC of 1.00, Bland–Altman analysis revealed significant variability (±27.9 PD), indicating challenges in clinical precision [18]. Despite these issues, its open-source design and remote functionality make it a promising tool for screening and triaging

Several non-VR objective systems have been developed to improve the standardization and efficiency of strabismus measurement. Video-oculography (VOG) systems have been shown to correlate well with manual prism testing [19,20,21]. A larger comparative study by Cantó-Cerdán et al. reported excellent agreement between VOG and APCT (R = 0.980 for esotropia and R = 0.975 for exotropia), with small mean differences and narrow limits of agreement in Bland–Altman analysis [19]. Earlier smaller studies also demonstrated reasonable accuracy and test–retest reliability using VOG combined with alternate cover testing [20,21]. In pediatric populations, eye-tracking-based tests (ETBT) showed better repeatability than manual PACT, with strong correlation and reliable performance even in children as young as 1.8 years [7]. Automated systems using full occlusion glasses and real-time eye tracking achieved high correlation (R > 0.9) with manual assessments and successfully classified phorias and tropias [8]. Additionally, telemedicine tools using video glasses enabled high agreement (ICC = 0.97, κ = 0.9–1.0) in remote pediatric strabismus evaluations [9]. Compared to these systems, our VR-based method integrates real-time eye tracking with immersive environments, offering additional potential for patient interaction and future therapeutic applications.

In our study, better correspondence to APCT was achieved with our CR method compared to the TO method (R^2^ = 0.934 vs. 0.874, respectively). However, in the subgroup analysis, exodeviation tended to be underestimated regardless of the method used. This phenomenon has also been observed in previous VR studies [10,12,14,22]. Potential explanations include the effects of accommodation to near targets displayed in VR systems, which may influence the AC/A ratio and lead to deviations in measurements. Additionally, intermittent exotropia, common in many patients, may introduce challenges in accurately capturing the deviated position due to the intermittent nature. An increased phoria difference between near and far distances under VR conditions has also been hypothesized as a contributing factor [12]. To address these limitations, further refinement of algorithms and improvements in system latency are needed to enhance the accuracy of VR-based measurements in exotropic cases.

The key advantage of our software lies in its implementation of the CR method. While the TO method is a straightforward and widely used approach, its limitations become evident when considering applications in immersive environments. TO adjusts only the position of the target, making it less suitable for scenarios requiring a dynamic and realistic visual experience. In contrast, the CR method shifts the entire visual scene, including the background and all objects within the environment. This comprehensive adjustment enables the creation of controlled and realistic simulations of visual conditions, which are essential for expanding VR applications beyond diagnostics. For example, immersive VR systems like the FDA-approved Luminopia, designed for amblyopia therapy, rely on dynamic and engaging visual environments to improve compliance and therapeutic outcomes [23]. Similarly, the CR method can facilitate the development of VR-based projects and interactive games tailored for strabismus training and rehabilitation. It serves as a foundational approach for future advancements in VR applications.

VR offers promising applications in the field of ophthalmology in the following aspects. First, VR technology can facilitate telemedicine by enabling remote monitoring and at-home assessments for patients with an established diagnosis of strabismus, reducing the need for frequent in-person visits [24,25]. Second, VR has the potential to address the global shortage and uneven distribution of pediatric ophthalmologists by serving as a community-based screening tool. This approach is particularly beneficial in rural areas with limited access to pediatric ophthalmologists. Similarly to autorefractor devices, VR systems could be used as a screening tool in schools or community centers to identify children requiring further evaluation. Lastly, VR-based systems could incorporate future therapeutic capabilities, offering a comprehensive solution for strabismus management [26]. Future VR platforms may combine ocular misalignment measurement with therapeutic interventions, providing accessible and integrated options for both diagnosis and treatment.

Several limitations should be addressed. The most obvious one is the relatively small sample size, which limits the external validity of our findings. However, our results align with previous reports, supporting the validity of our study. Second, commercial VR headsets may be too large for young children, potentially causing bias and incorrect data. We tried to eliminate this bias by selecting children whose body size was more comparable to that of adults. This also raises concerns about the overall feasibility and compliance of VR-based tests in younger pediatric populations. Ocular deviation was only tested in the primary position at a distance, with only horizontal deviations recorded and no intra- or inter-observer comparisons made. In addition, the generalizability of our results to other types of strabismus, such as vertical or incomitant deviations, remains uncertain. Further VR designs should include cyclotorsion, vertical deviation measurements, and near tests at 0.33 m to fully evaluate the patient’s oculomotor status. Cybersickness, which presents as motion sickness-like symptoms, is another concern with prolonged immersion in a VR environment. Although our questionnaire revealed no discomfort after the tests, it is still worth considering if VR is used long-term. Also, the present study did not include a control group of healthy individuals. Including such a group in future studies would allow for assessment of system specificity and help define normative deviation thresholds for clinical use.

In conclusion, this newly developed VR system software, incorporating the CR method, may offer a feasible approach for measuring ocular deviation. The CR method showed stronger correlation with APCT than the previously established TO method. By shifting the entire visual scene rather than just the target, it may support the development of future VR applications in immersive environments. Further refinements and larger-scale validation studies are needed to confirm its clinical usefulness.

## Figures and Tables

**Table 1:** Demographic and Clinical Characteristics of the Subjects.

Age in Years, Mean (Range), Median (IQR)	22.3 (9–46), 21.5 (17–25)
Female (%)	77.8%
Measurements obtained in glasses *n* (%)	12, (61.1%)
Manifest strabismus, *n* (%)	5, (27.8%)
Esotropia, *n* (%)	6, (33.3%)
Exotropia, *n* (%)	12, (66.7%)

**Table 2:** Deviation Angle Measurements by APCT and VR Using Two Methods.

**Methods**	**Number**	**Deviation Angle (PD)**	***p*** **Value**
APCT	18	16.00 ± 32.23	
TO	18	7.67 ± 29.54	0.604
CR	18	6.22 ± 32.36	
**Subgroup**	**Methods**	**Paired Difference in Deviation Angle (PD)**	***p*** **Value**
Esotropia *n* = 6	TO-CR	5.12 ± 11.69	0.328
	APCT-TO	3.83 ± 11.44	0.499
	APCT-CR	9.00 ± 9.38	0.066
Exotropia *n* = 12	TO-CR	−0.43 ± 5.88	0.811
	APCT-TO	10.58 ± 11.23	0.008 ^1^
	APCT-CR	10.17 ± 8.19	0.001 ^1^

^1^ *p* < 0.05. APCT: alternative prism cover test, TO: target offset CR: camera rotate, PD: prism diopters, The deviation angle is shown as mean ± standard deviation.

## Data Availability

The original contributions presented in this study are included in the article. Further inquiries can be directed to the corresponding authors.

## Abbreviations

The following abbreviations are used in this manuscript:

VR	Virtual Reality
XT	exotropia
ET	esotropia
APCT	alternate prism cover test
TO	target offset
CR	camera rotation
VA	visual acuity
PD	prism diopters
